# Erythrocyte Transketolase Activity, Markers of Cardiac Dysfunction and the Diagnosis of Infantile Beriberi

**DOI:** 10.1371/journal.pntd.0000971

**Published:** 2011-02-22

**Authors:** Douangdao Soukaloun, Sue J. Lee, Karen Chamberlain, Ann M. Taylor, Mayfong Mayxay, Kongkham Sisouk, Bandit Soumphonphakdy, Khaysy Latsavong, Kongsin Akkhavong, Douangkham Phommachanh, Vanmaly Sengmeuang, Khonsavanh Luangxay, Theresa McDonagh, Nicholas J. White, Paul N. Newton

**Affiliations:** 1 Wellcome Trust-Mahosot Hospital-Oxford Tropical Medicine Collaboration, Microbiology Laboratory, Mahosot Hospital, Vientiane, Lao People's Democratic Republic; 2 Centre for Tropical Medicine, Churchill Hospital, Nuffield Department of Medicine, University of Oxford, Oxford, United Kingdom; 3 Faculty of Tropical Medicine, Mahidol University, Bangkok, Thailand; 4 MRC Human Nutrition Research, Cambridge, United Kingdom; 5 Faculty of Postgraduate Studies, University of Health Sciences, Vientiane, Lao People's Democratic Republic; 6 Royal Brompton Hospital, London, United Kingdom; The George Washington University Medical Center, United States of America

## Abstract

**Background:**

Infantile beriberi is a potentially lethal manifestation of thiamin deficiency, associated with traditional post-partum maternal food avoidance, which persists in the Lao PDR (Laos). There are few data on biochemical markers of infantile thiamin deficiency or indices of cardiac dysfunction as potential surrogate markers.

**Methodology/Principal Findings:**

A case control study of 47 infants with beriberi and age-matched afebrile and febrile controls was conducted in Vientiane, Laos. Basal and activated erythrocyte transketolase activities (ETK) and activation (α) coefficients were assayed along with plasma brain natriuretic peptide, N-terminal pro-brain natriuretic peptide and troponin T. Basal ETK (and to a lesser extent activated ETK) and plasma troponin T were the only infant biochemical markers that predicted infantile beriberi. A basal ETK≤0.59 micromoles/min/gHb gave a sensitivity (95%CI) of 75.0 (47.6 to 92.7)% and specificity (95%CI) of 85.2 (66.3 to 95.8)% for predicting infantile beriberi (OR (95%CI) 15.9 (2.03–124.2); p = 0.008) (area under ROC curve = 0.80). In contrast, the α coefficient did not discriminate between cases and controls. Maternal basal ETK was linearly correlated with infant basal ETK (Pearson's r = 0.66, p<0.001). The odds of beriberi in infants with detectable plasma troponin T was 3.4 times higher in comparison to infants without detectable troponin T (OR 3.4, 95%CI 1.22–9.73, p = 0.019). Detectable troponin T had a sensitivity (95%CI) of 78.6 (59.0 to 91.7) % and specificity (95%CI) of 56.1 (39.7 to 71.5) % for predicting infantile beriberi.

**Conclusions/Significance:**

Basal ETK is a more accurate biochemical marker of infantile beriberi than the activation coefficient. Raised plasma troponin T may be a useful indicator of infantile beriberi in infants at risk and in the absence of other evident causes.

## Author Summary

Infantile beriberi, or clinical thiamin (vitamin B1) deficiency in infants, is a forgotten disease in Asia, where ∼100 years ago it was a major public health problem. Children aged ∼2–3 months present in cardiac failure but usually rapidly improve if given thiamin injections. It remains relatively common in Vientiane, Lao PDR (Laos) probably because of prolonged intra- and post-partum maternal food avoidance behaviours. There has been very little recent research on the best diagnostic techniques. We conducted a case control study of 47 infants with beriberi and age-matched afebrile and febrile controls in Vientiane. The conventional measures of thiamin deficiency, basal and activated erythrocyte transketolase activities (ETK) and activation (α) coefficients, were assayed along with three markers of cardiac dysfunction - plasma brain natriuretic peptide, N-terminal pro-brain natriuretic peptide, and troponin T. Basal ETK was a better biochemical marker of infantile beriberi than the activation coefficient. Raised plasma troponin T may be a useful indicator of infantile beriberi in babies at risk and in the absence of other evident causes.

## Introduction

Infantile beriberi, or clinical thiamin deficiency in infants, was first described in Japan by Hirota in 1898 [1]. With the advent of mechanical rice milling in the late 19^th^ century, which removed the main dietary source of thiamin in rice husk, beriberi became a major public health problem in Asia, responsible for considerable mortality [1]–[4]. In the early 20^th^ century there was an enormous international effort to understand the causes, epidemiology and prevention of this devastating and common disease in the rice consuming societies of Asia [1]–[9]. Thiamin deficiency may present with a variety of clinical syndromes including encephalopathy, hypoglycemia and lactic acidosis, but the predominant overt presentation is myocardial dysfunction [10]. Infantile beriberi typically presents as shock, often preceded by a hoarse cry, in babies of approximately three months of age [7], [8].

With the identification of the aetiology of beriberi, changes in diet, supplementation and targeted health programmes to ensure adequate thiamin intake, the incidence of, and interest in, this disease in the more accessible, wealthier parts of Asia declined markedly. However, there have been recent reports suggesting that infantile beriberi does remain an important public health problem in Asia [10]–[15].

In Laos, beriberi was acknowledged as an important disease in the 1920s [16], in the 1960s and 1970s [15], [17], [18] and was rediscovered in the early 1990s as a cause of infant death in the capital Vientiane [15]. Some 50–90 infants with a clinical diagnosis of beriberi are admitted each year to Mahosot Hospital, Vientiane [19].

The period of intensive research on infantile beriberi occurred before the invention of the erythrocyte transketolase activity (ETK) assay [20] and there have been very few investigations of infantile beriberi in the last 40 years [10]–[15], [21]–[23]. Thiamin deficiency is conventionally assessed using functional assays for the thiamin-dependent ETK enzyme in *ex vivo* washed red cells. The activation coefficient (α) is the ratio of *in vitro* ETK after thiamin pyrophosphate has been added (activated ETK) minus the basal ETK before thiamin pyrophosphate has been added, to the basal ETK, expressed as a percentage. Higher α represent greater thiamin deficiency. However, few laboratories are able to perform the assays and the tests are expensive. Importantly, the test and appropriate cutoffs for α have only been evaluated in adults with beriberi [24]–[27], usually in non-rice eating societies. Whether reference ranges for α vary by geography, ethnicity and diet is uncertain. There have been only five reports of ETK determinations in infants older than one week. Pongpanich *et al.* [28] showed in three patients with beriberi that α was high and basal ETK low but no details of the children were given. Tanphaichtr [29] reported a mean α in 9 infants with beriberi of 34% but no other details were provided. Debuse [30] described an Australian infant with beriberi with low basal ETK and α of 27% but no assay details were given. Migasena *et al.* [31] measured α in Thai maternal and newborn blood, finding that 42% of mothers but only 2% of newborns were deficient. In an epidemic of infantile beriberi in Israel, linked to a thiamin-deficient formula, α was >15% in 8/9 infants [21]. Among the mothers of 45 Burmese infants with clinical beriberi, 53% had α>20% with mean α of 23.3%, in comparison to mean α of 8.2% for 57 mothers of infants without clinical beriberi [10].

There is evidence that the activity of the ETK apoenzyme may decrease in the relative absence of thiamin and although basal ETK is reduced, α may be normal. Therefore, α may not be the appropriate measure of thiamin deficiency in young infants as, with very low exposure to thiamin *in utero* and post-partum, the infant ETK apoenzyme may be unstable and concentrations fall, underestimating the frequency of biochemical thiamin deficiency [3], [4], [24]–[25], [30], [32]. If Lao mothers and infants, with many risk factors for thiamin deficiency, are exposed to chronically low thiamin concentrations for all their lives, including *in utero*, basal ETK, rather than the α, may be the better measure.

In addition to the uncertainty of appropriate ETK assessment of infantile beriberi, there is a need for more straightforward and inexpensive tests to evaluate thiamin deficiency. Classical infantile beriberi may be easy to recognize but it is likely that in chronically thiamin deficient societies, infants may present with other thiamin deficient syndromes and that the clinical features of beriberi may be obscured by other precipitating diseases [19]. As heart failure is a consistent feature of ‘wet’ beriberi, recently described circulating markers of cardiac dysfunction, such as brain natriuretic peptide (BNP), N-terminal pro-brain natriuretic peptide (ntBNP) and troponin T [33], are likely to be elevated in infantile beriberi and could, in the absence of confounding diseases, suggest a diagnosis of beriberi. We therefore conducted a prospective case control study to investigate ETK assays of maternal and infant venous blood. We examined the relationship between basal ETK and α, and markers of cardiac dysfunction in infants with clinical beriberi in comparison to febrile and afebrile age matched controls.

## Methods

### Ethics

Ethical approval for the study was granted by the Ministry of Health, Government of the Lao PDR. Infants and their mothers were included if the mothers gave witnessed informed oral consent. The IRB approved the use of oral consent, which was thought to be the most appropriate mode for this study. Mothers were given an information sheet describing the study and informed oral consent was documented by the signature of someone who was not a member of the study team.

### Patients

Infants (<1 year old) suspected of having infantile beriberi by admitting paediatricians at Mahosot Hospital, Vientiane, were included consecutively when clinical duties permitted, in 2001–2005, provided that the mother/guardian gave informed oral consent. Suspected infantile beriberi was defined as one or more of the following: shock with BP systolic <70 mmHg or a pulse pressure <20 mmHg, peripheral or central cyanosis, tachycardia >130/min or bradycardia <80/min or grunting, without fever (<37.5°C) and other known causes of these physical signs. For each infant-mother pair recruited, two age-matched control infants, without clinical evidence of beriberi, and their mothers were recruited from the same hospital provided the mothers gave informed oral consent. The two control groups consisted of the next febrile (≥37.5°C) infant of the same age (±one month) admitted to the same ward and the next sick afebrile (<37.5°C) infant of the same age (±one month) admitted to the general paediatric and paediatric surgical wards or seen at the Mother and Child Health Clinic. We recruited two control groups to allow comparison of infants with beriberi with those without clinical evidence of beriberi but with and without fever. We aimed to study 50 cases and 100 controls. The medical history of the infant and mother, the mother's food taboos, economic status and the infant's diet were recorded for cases and both controls (Tables 1 & 2). Blood (1.8ml) was collected into lithium heparin tubes, from both infant and mother when possible.

**Table 1:** Characteristics of infants with beriberi and febrile and afebrile controls.

Variable	Clinical beriberi	Febrile controls	Afebrile controls	P^e^
**Infants**	4717	4717	4712	
Age/months^a^	2.0 (0.8–8)2.0 (0.8–3)	1.9 (0.8–11)1.2 (0.8–4)	2.0 (0.3–9)^46^1.2 (1–3.4)	0.70.2
Male %	29/47 (62)10/17 (59)	26/47 (56)10/17 (59)	26/47 (56)7/12 (58)	0.51.0
Sibling died <1 year old^c^	11/42 (26.2)11/15 (73.3)	0/42 (0)0/17 (0)	3/36 (8.3)4/7 (57.1)	0.0050.2
Breast fed %	46/46 (100)17/17 (100)	40/44 (90.9)16/17 (94.1)	42/45 (93.3)9/11 (81.8)	---g---g
Bottle fed %	1/46 (2.2)0/17 (0)	13/44 (29.6)4/17 (23.5)	11/45 (24.4)4/11 (36.4)	0.010.01
Given rice water %	4/46 (8.7)2/17 (11.8)	7/44 (15.9)1/17 (5.9)	9/45 (20.0)3/11 (27.3)	0.20.6
Given chewed sticky rice %	12/46 (26.1)3/17 (17.7)	8/44 (18.2)1/17 (5.9)	5/45 (11.1)0/11 (0)	0.070.1
Peripheral White cell count 10^9^/L^a^	11.0 (6.0–33.0)^35^10.0 (6.1–13.0)^6^	11.4 (4.5–28.9)^28^9.0 (5.8–12.2)^2^	11.0 (6.8–30.5)^31^9.8 (7.3–11.0)^3^	1.0---
Hct %^a^	34 (16–50)^34^33 (24–42)^6^	35 (22–55)^28^35.0 (30–40)^2^	34 (17–50)^31^40 (34–50)^3^	1.0---
Basal erythrocyte transketolase activity micromoles/min/gHBb,	0.53 (0.47–0.60)^17^	0.78 (0.65–0.91)^17^	0.75 (0.61–0.89)^12^	0.02
Basal erythrocyte transketolase activity <0.59 micromoles/min/gHB	13/17 (76.5)	4/17 (23.5)	3/12 (25)	0.001
Activated erythrocyte transketolase activity micromoles/min/gHB^b^	0.62 (0.53–0.70)^17^	0.88 (0.73–1.04)^17^	0.85 (0.72–0.98)^12^	0.02
Alpha %^a^	18.2 (−9 to 625)^42^10.1 (−5.4–72.4)^17^	13.0 (−2.5–558.3)^40^10.3 (−2.5–73.7)^17^	11.8 (−35.7–779.9)^38^12.8 (4.2–33.9)^12^	0.50.9
Alpha ≥25%	16/42 (38.1)3/17 (17.7)	13/40 (32.5)3/17 (17.7)	9/38 (23.7)3/12 (25)	0.30.6
Alpha >31%	13/42 (30.9)2/17 (11.8)	10/40 (25)1/17 (5.9)	5/38 (13.2)1/12 (8.3)	0.20.5
BNP pg/ml^a^	334 (0–2,465)^38^158 (2–1,978)^17^	7 (0–590)^29^8 (0–361)^13^	6 (0–169)^30^6 (0–169)^12^	0.080.0005
% with BNP>upper limit reference range for age/sex^d^	9/38 (23.7)2/17 (11.8)	0/290/13	0/300/12	---h---h
NT-pro-BNP pg/ml^a^	35,000 (128–>35,000)^15^32,080 (128–35,000)^13^	1,514 (335–6,831)^10^1,514 (335–6,831)^10^	866 (96–35,000)^11^866 (96–35,000)^11^	0.030.0009
% with NT-pro-BNP >650 pg/ml	14/15 (93)12/13 (92.3)	6/12 (50)8/10 (80)	9/11 (82)6/11 (54.6)	0.20.09
% with NT-pro-BNP >upper limit reference range for age/sex^d^	11/15 (73.3)9/13 (69.3)	0/100/10	1/11 (9.1)1/11 (9.1)	<0.001<0.001
Troponin T ng/ml^a^	0.07 (0–0.893)^35^0.11 (0–0.351)^14^	0 (0–0.159)^27^0 (0–0.052)^11^	0 (0–0.122)^30^0 (0–0.058)^12^	0.0050.003
% with detectable troponin T	27/35 (77.1)11/14 (78.6)	9/27 (33.3)4/11 (36.4)	14/30 (46.7)8/12 (66.7)	0.0190.1

Superscript numbers refer to sample size if data missing.

a median (range),

b mean (95%CI),

c of those with siblings,

d for reference ranges see table base,

e matched comparison of both control groups combined, with cases, except

f matched comparison of the groups separately, the first P value refers to cases versus febrile controls and the second P value to cases versus afebrile controls,

g p-value could not be obtained in the matched analysis as the mothers of cases were all breast fed,

h p-value could not be obtained in the matched analysis as the results for all controls were zero.

**d**

97.5th percentiles for males and females over the age range 0–1 years for

BNP (pg/ml)

0–<31 days M and F 1,585

31–<90 days M and F 1,259

3–<6 months M and F 759

6 months–<1 y M and F 263

97.5th percentiles for males and females over the age range 0–1 y for NT-pro-BNP

(pg/ml) [41]

0–<31 days M 28,184

0–<31 days F 35,481

31–<90 days M 19,953

31–<90 days F 15,135

3–<6 months M 15,849

3–<6 months F 14,125

6 months–<1 y M 11,220

6 months–<1 y F 10,000

The first line in each row refers to all those with data and the second line to only infants with basal ETK data.

**Table 2:** Characteristics of mothers and fathers of infants with beriberi and febrile and afebrile controls.

Variable	Clinical beriberi	Febrile controls	Afebrile controls	P^e^
**Mothers**	4717	4717	4712	
Age/years^a^	24 (15–47)24 (18–37)^17^	25 (16–43)25 (16–38)^17^	25 (18–39)26 (19–39)^12^	0.70.5
Lao Loum ethnic group^i^	35/47 (74.5)12/17 (70.6)	39/47 (83.0)16/17 (94.1)	43/47 (91.5)11/12 (91.7)	0.070.05
% of mothers' rice farmers	20/47 (42.6)11/17 (64.7)	13/46 (28.3)4/17 (23.5)	7/47 (14.9)2/12 (16.7)	0.0180.004
Home in Vientiane City	36/47 (76.6)12/17 (70.1)	41/47 (87.2)15/17 (88.2)	43/47 (91.5)10/12 (83.3)	0.040.18
Gravidity^a^	2 (1–11)2 (1–7)^17^	2 (1–6)2 (1–4)^15^	2 (1–8)2 (1–8)^12^	0.10.7
Schooling/years^a^	5 (0–11)5 (0–11)^17^	5 (0–16)5 (0–16)^17^	8 (0–14)8 (0–14)^12^	0.37 0.001^f^0.10
Enough money for food	35/39 (89.7)13/17 (76.5)	35/38 (92.1)14/17 (82.4)	40/45 (88.8)9/12 (75.0)	0.90.6
Observed food avoidance	43/47 (91.5)16/17 (94.1)	22/47 (46.8)8/17 (47.1)	35/46 (76.1)10/12 (83.3)	0.001 0.07^f^0.06
Parasthaesia	30/47 (63.8)8/18 (47.1)	1/19 (5.3)1/16 (6.3)	5/19 (26.3)3/12 (25.0)	0.0260.02
Peripheral White cell count 10^9^/L^a^	10.1 (5.8–20.0)^30^7.5 (5.8–9.2)^2^	9.6 (5.0–23.4)^27^7.9^1^	9.8 (5.8–20.0)^28^----^0^	0.23-----
Hct %^a^	35 (22–46)^30^33 (31–35)^2^	38 (27–45)^27^38^1^	35 (25–45)^28^---^0^	0.8---
Basal erythrocyte transketolase activity micromoles/min/gHb^a^^,^ d	0.54 (0.23–0.90)^17^	0.72 (0.37–1.3)^17^	0.58 (0.38–0.95)^12^	0.04
Activated erythrocyte transketolase activity micromoles/min/gHb^a^	0.67 (0.29–0.94)^17^	0.79 (0.48–1.42)^17^	0.68 (0.48–1.02)^12^	0.05, 0.72^f^
% with basal erythrocyte transketolase activity micromoles/min/gHb <lower limit reference range for age^d^	9/17 (52.9)	2/17 (11.8)	4/12 (33.3)	0.037
Alpha %^a^	20.3 (−17.4–273.9)^40^23.4 (−2.8–52.0)^16^	11.9 (−32.1–360.5)^39^9.5 (−2.3–41.4)^17^	15.3 (−31.2–466.1)^36^10.4 (1–41.3)^12^	0.50.09
Alpha ≥25%	17/40 (42.5)8/16 (50.0)	10/39 (25.6)3/17 (17.7)	15/36 (41.7)4/12 (33.3)	0.250.08
Alpha >31%	11/40 (27.5)4/16 (25.0)	7/39 (18)2/17 (11.8)	7/36 (19.4)2/12 (16.7)	0.160.3
BNP pg/ml^a^	4 (0–203)^41^2 (0–203)^17^	3 (0–64)^4^4 (0–26)^14^	4 (0–101)^29^3 (0–23)^12^	0.60.5
% with BNP≥100 pg/ml	1/43 (2)1/17 (5.9)	0/33 (0)0/14 (0)	1/32 (3)0/12 (0)	0.80.4
NT-proBNP pg/ml^a^	57 (11–14,492)^18^57 (11–14,492)^16^	38 (14–198)^12^38 (14–198)^12^	37 (10–146)^11^37 (10–146)^11^	0.30.2
% with NT-proBNP ≥300 pg/ml	3/19 (16)2/16 (12.5)	0/12 (0)0/12 (0)	0/12 (0)0/11 (0)	---h--h
Troponin T ng/ml^a^	0 (0–0.319)^40^0 (0–0.319)^16^	0^30^0^14^	0^29^0^12^	--h--h
% with troponin T >0.03 ng/mL	1/40 (2.5)1/16 (6.3)	0/30 (0)0/14 (0)	0/29 (0)0/12 (0)	--h--h
**Fathers**				
Father cash expenditure/day in kip^a^	8,000 (0–100,000)^19^----^0^	10,000 (0–50,000)^23^----^0^	15,000 (0–200,000)^25^----^0^	0.97---
% fathers' rice farmers	27/47 (57.5)12/17 (70.6)	14/45 (31.1)6/17 (35.3)	6/44 (13.6)4/12 (33.3)	<0.0010.019

Superscript numbers refer to sample size if data missing.

a median (range),

b mean (95%CI),

c of those with siblings,

d for reference ranges see table base,

e matched comparison of both control groups combined, with cases, except

f matched comparison of the groups separately, the first P value refers to cases versus febrile controls and the second P value to cases versus afebrile controls,

g p-value could not be obtained in the matched analysis as the mothers of cases were all breast fed,

h p-value could not be obtained in the matched analysis as the results for all controls were zero,

i ‘Lao Loum’ or ‘lowland Lao’ or ‘Tai-Kadai ethno-linguistic family’ see Ref [54].

d Basal erythrocyte transketolase activity (unpublished data NDNS Young people and Adult surveys, UK)

For females aged/years micromoles/min/gHb

4–18 ≤0.57

19–24 ≤0.57

25–34 ≤0.50

35–49 ≤0.47

The first line in each row refers to all patients with data and the second line to only those with infant basal ETK data.

### Laboratory investigations

#### a) Erythrocyte transketolase

Immediately after collection, lithium heparin anticoagulated blood was centrifuged and washed in phosphate buffered saline three times, with removal of the buffy coat initially and after each wash. Washed red cells were stored at −30°C for a maximum of 3 months and then at −70°C until shipment to the UK on dry ice. Due to retirement of the machine in the first laboratory, ETK assays were performed at two laboratories [19], blinded to the results of the cardiac marker assays (below). In Oxford (patient triplets 1–28) the assay was performed by a modification of the nicotinamide-adenine dinucleotide dependent method with ribose-5-phosphate as the substrate [34], [35] except that samples were collected into acid citrate dextrose in [35]. In Cambridge an adaptation of the method of Vuilleumier *et al.* [36] was used (patient triplets 29–50). A Cobas Fara (Roche Co., Gipf-Oberfrick, Switzerland) was used in both laboratories and at both sites α was calculated as: ((ETK Activated - ETK basal)/ETK Basal)×100. Haemoglobin concentrations were not assayed in Oxford and therefore basal ETK could not be expressed for these 28 sample triplets in micromoles/min/gHb. The coefficients of variation for the ETK assays in Oxford and Cambridge were 9.0–10.0 and 5.1–7.7, respectively. There are few data from which to determine the reference range for basal ETK. ETK has been shown to decline with increased age but using a different technique to that used here [37], [38]. For the reference range for maternal basal ETK we used unpublished data from the NDNS Young people and Adult surveys, UK (MRC Human Nutrition Research unpublished, Table 2). We have found no comparable data for Asian adult females, nor have we found data describing the reference range for ETK in infants in which comparable techniques have been used. Different upper reference ranges for α are described and we have used ≥25% and the more conservative α of >31% [39] as defining definite severe biochemical thiamin deficiency. Storage of *ex vivo* human washed red cells for 18 months at −70°C has been shown not to affect α values [35], [39].

#### b) Cardiac markers

Markers of cardiac dysfunction assayed were BNP (IRMA BNP kits Cis Bio International, France), NT pro-BNP (hereafter ntBNP; Elecsys proBNP, Roche, Basel, Switzerland) and troponin T (Elecsys 2010, Roche, Basel, Switzerland). The investigator performing these assays was blinded to the results of the ETK assays. For BNP we used upper limit values of ≥100 pg/ml for mothers [40] and the age-specific 97.5th percentiles for children from Soldin *et al.* [41] (see Tables 1 & 2). For NT-pro-BNP we used upper limit values of ≥300pg/ml for mothers [41] and 650 pg/ml for children aged 1 month-1 year [42] and the age-specific 97.5th percentiles for children from Soldin *et al.* [41] (see Tables 1 & 2). In infants any detectable troponin T (lower limit of detection = 0.01ng/mL) [43] and in mothers >0.03 ng/mL [44] were regarded as significant. Lithium heparin anticoagulated plasma, removed after the first centrifugation in the preparation of washed red cells, was used for these assays. Infants with beriberi were given parenteral thiamin (50–100 mg) as soon as possible, followed by parenteral/oral thiamin for both infant and mother.

### Statistical analysis

To establish which biochemical markers predicted infantile beriberi, conditional logistic regression, with a variable to identify each matched set, was used [45]. Receiver operating characteristic (ROC) curves were used to determine optimal cut-off ranges for any continuous biochemical markers that did not have previously published or accepted ranges. Predictive ability was assessed further by calculating sensitivities and specificities. Similarly, to identify which clinical factors were associated with infantile beriberi, conditional logistic regression for matched data was used. Although the primary objective was to determine if cases differed from controls, the secondary objective was to determine whether cases differed from febrile controls and whether cases differed from afebrile controls. Therefore, the McNemar's test was used to assess whether febrile controls were significantly different from afebrile controls. If they were different, the comparison against cases was performed separately for each control group. Because of multiple comparisons, a Bonferroni adjusted p-value conservatively rounded down to 0.02 was used. Any significant factors were then included in a multivariate model to identify independent predictors of infantile beriberi. Using a stepwise selection procedure, only variables that were significant at P<0.05 were retained in the final model. Using these independent clinical predictors, a separate model for each biochemical marker that was identified as a significant predictor of beriberi was run to confirm its predictive ability in an adjusted model. All analyses were conducted using Stata (v10, StataCorp., College Station, Texas). We have attempted to report the study according to the STARD guidelines [46].

## Results

Fifty patients in each of the three groups were recruited. However, 3 of the cases did not fulfill the criteria for infantile beriberi (above) and therefore these infants, their mothers and their controls were not included, leaving 47 matched sets for analysis.

### Clinical features

The admission clinical features of the 141 infants and their parents are shown in Tables 1 & 2. Additional admission information was collected for infants with clinical beriberi-these infants had lethargy (74%), poor feeding (72%), vomiting (60%), dyspnoea (81%), grunting (82%, n = 45), palpable liver (96%, n = 27), peripheral oedema (25%, n = 44), cold fingers (76%, n = 46), central cyanosis (66%, n = 18), peripheral cyanosis (44%), median (range) pulse 140 (95–175)/min (n = 42), mean (95%CI) respiratory rate 46.0 (42.7–49.3)/min (n = 40), and mean (95%CI) temperature 36.6 (36.3–36.8)°C (n = 43). For mothers of cases, mean (95%CI) respiratory rate, pulse and systolic blood pressure were 22.5 (20.4–24.5)/min (n = 40), 87.0 (82.4–91.5)/min (n = 40) and 107 (103–110) mmHg (n = 37), respectively. Mothers of cases had peripheral oedema in 4/45 (8.9%) and tender soles in 21/28 (75%). Three of the infant cases died.

### Which biochemical markers best predict clinical infantile beriberi?

Using continuous measures or previously published cut-offs, infant basal ETK, activated ETK and plasma troponin T were the only infant biochemical markers that predicted infantile beriberi consistently (Table 1).

Mean (95%CI) infant basal ETK was lower in cases (0.53 (0.47–0.60) micromoles/min/gHb, n = 17), than in those without beriberi (0.77 (0.68–0.86), n = 29, p = 0.02)–in those with fever (0.78 (0.65–0.91, n = 17) micromoles/min/gHb, with mean (95%CI) control-case difference = 0.284 (0.133–0.435) micromoles/min/gHb) and those without fever (0.75 (0.61–0.89, n = 12) micromoles/min/gHb, with mean (95%CI) control-case difference = 0.218 (0.039–0.397) micromoles/min/gHb). Mean (95%CI) infant activated ETK was lower in cases (0.62 (0.53–0.70, n = 17) micromoles/min/gHb), than in those without beriberi (0.87 (0.77–0.97), n = 29, p = 0.02)-in those with fever (0.88 (0.73–1.04, n = 17) micromoles/min/gHb, with mean (95%CI) control-case difference 0.301 (0.120–0.481) micromoles/min/gHb) and those without fever (0.85 (0.72–0.98, n = 12) micromoles/min/gHb, with mean (95%CI) control-case difference 0.231 (0.062–0.400) micromoles/min/gHb).

There were 16 matched sets (43 observations) with at least one control with basal ETK data available. Since no reference ranges of basal and activated ETK have been defined for infants, ROC analysis was performed to find the optimum cut-offs. Basal ETK<0.59 micromoles/min/gHb gave a sensitivity (95%CI) of 75.0 (47.6 to 92.7)% and specificity (95%CI) of 85.2 (66.3 to 95.8)% for predicting beriberi. The odds of having beriberi were 15.9 times higher (95%CI 2.03 to 124.2; p = 0.008, area under ROC curve = 0.80) for infants with basal ETK<0.59 micromoles/min/gHb when compared with patients with basal value ≥0.59. Activated ETK≤0.74 micromoles/min/gHb gave a sensitivity (95%CI) of 75.0 (47.6 to 92.7)% and specificity (95%CI) of 74.1 (53.7 to 88.9)%. The odds of having beriberi for infants with an activated ETK<0.74 micromoles/min/gHb were 12.5 times higher (95%CI 1.58–99.3; p = 0.02, area under ROC curve = 0.75) when compared with patients with activated ETK≥0.74. The relationship between clinical beriberi and basal ETK was therefore stronger than with activated ETK.

In contrast, the infant α coefficient did not discriminate between cases and controls (OR 1.0 95%CI 0.997–1.01; n = 40 matched sets providing 112 observations for analysis). Using α≥25% or >31% to define biochemical thiamin deficiency were likewise not useful for predicting infantile beriberi (OR 1.48, 95%CI 0.66–3.31 and OR 1.90, 95%CI 0.77–4.70, respectively). With α coefficient as a continuous variable, ROC analysis identified α≥16.5% as the optimum cut-off (OR 2.4, 95%CI 1.06 to 5.44).

The continuous measure for ntBNP was borderline associated with beriberi (OR 1.00, 95%CI 1.000009–1.0002), but this analysis included only 11 matched sets with at least one control (n = 28 observations). The median (range) plasma ntBNP in beriberi infants (35,000 (128 to ≥35,000) pg/ml) was higher than in those without beriberi (1,049 (96 to 35,000) pg/ml). Using the Soldin et al. [41] cutoffs for ntBNP, eleven (73.3%) infants with beriberi, but only one of the controls (4.8%), had raised ntBNP. Prediction using these ntBNP cut-offs gave a sensitivity (95%CI) of 63.6 (30.8 to 89.1) % and specificity (95%CI) of 94.1 (71.3 to 99.9) %. Using a ntBNP cut-off of >650 pg/ml, the odds (95%CI) of having beriberi were 4.5 (0.47 to 42.7).

**Figure 1:**
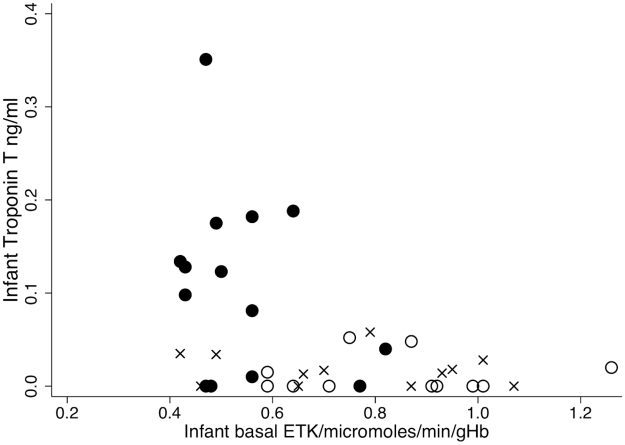
Relationship between infant basal ETK and infant Troponin T. Black circles = infants with beriberi (n = 14), crosses = infant afebrile controls (n = 12), open circles = infant febrile controls (n = 11).

The median (range) plasma BNP in beriberi infants (334 (0–2,465) pg/ml) was higher than in those without beriberi (7 (0–590) pg/ml) (OR 1.02 95%CI 1.00–1.05, P = 0.08; n = 31 matched sets providing 78 observations for analysis). Nine (23.7%) infants with beriberi, but none of the control infants, had raised BNP [41]. Using these cutoffs for raised BNP, as defined by Soldin et al. [41], the specificity (95%CI) was 100 (92.5 to 100) % but the sensitivity (95%CI) was low at 25.8 (11.9 to 44.6) %. However, the odds of beriberi in infants with detectable plasma troponin T were 3.4 times higher when compared with infants without detectable troponin T (OR 3.4, 95%CI 1.22 to 9.73, P = 0.019, n = 28 matched sets providing 69 observations). Detectable troponin T had a sensitivity (95%CI) of 78.6 (59.0 to 91.7) % and specificity (95%CI) of 56.1 (39.7 to 71.5) % for predicting infantile beriberi. Infants with low basal ETK tended to have higher troponin T (Spearman's rho = −0.39, p = 0.02, Fig. 1).

**Figure 2:**
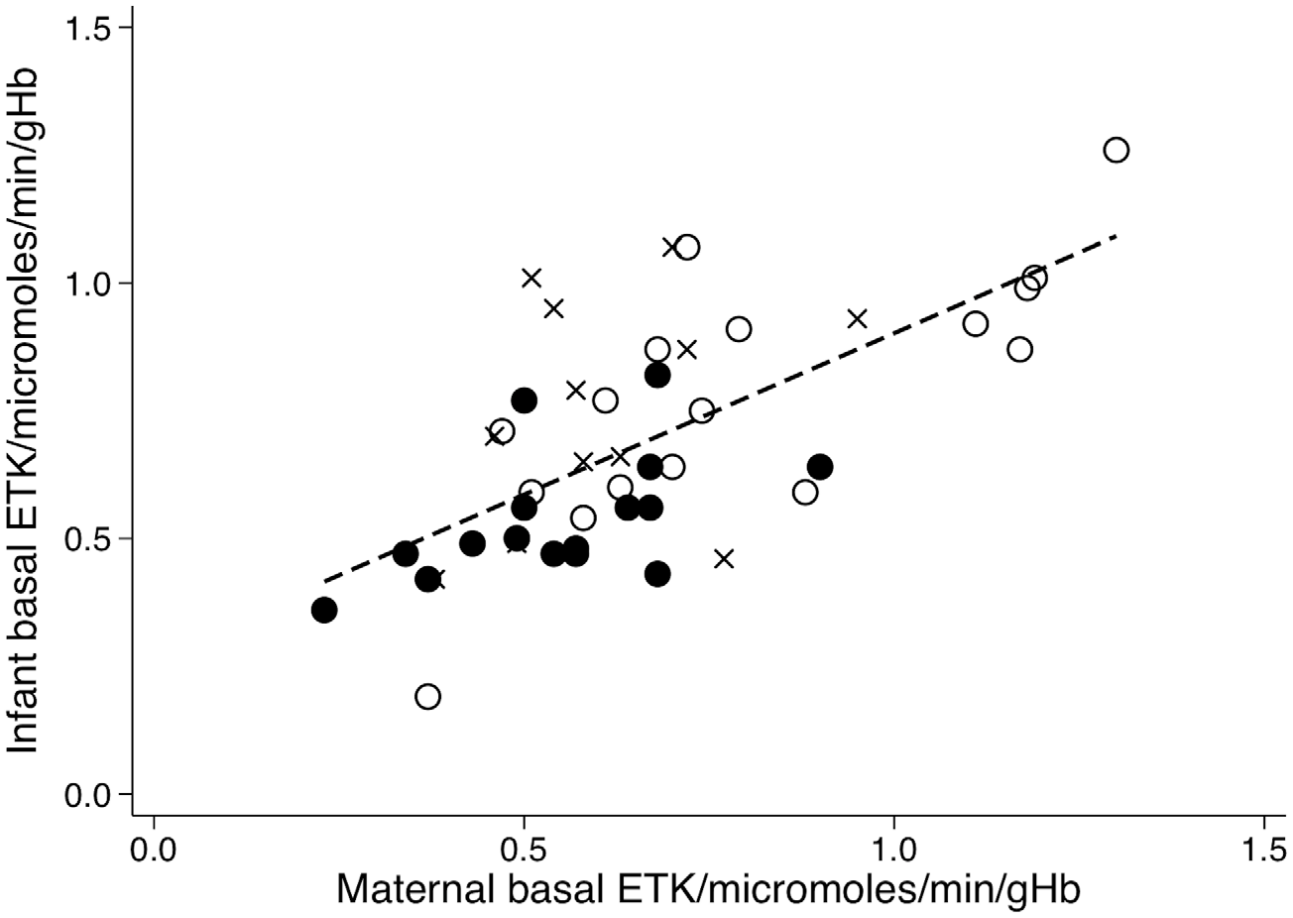
Relationship between maternal and infant basal ETK for 48 infant-mother pairs. Black circles = infant-mother pairs – infant with beriberi (n = 16), crosses = infant-mother pairs – afebrile controls (n = 12), open circles = infant-mother pairs – febrile controls (n = 17). Pearson's r = 0.66, P<0.001, Maternal basal ETK = 0.2699+0.6320*Infant basal ETK.

A low maternal basal ETK (<lower limit reference range for age, Table 2) was significantly associated with infantile beriberi (OR 5.4, 95%CI 1.11 to 26.0, n = 17 matched sets giving 45 observations for analysis). However, maternal activated ETK (as a continuous variable) was only borderline associated with infantile beriberi, with very wide CIs (OR 18.8, 95%CI 0.85 to 416, n = 17 matched sets giving 45 observations for analysis, P = 0.06). Maternal basal ETK was significantly linearly correlated with infant basal ETK (Pearson's r = 0.66, p<0.001) (Fig. 2). The maternal α coefficient did not discriminate between cases and controls (OR 1.0 95%CI 0.995–1.01; n = 40 matched sets providing 108 observations for analysis). Maternal α≥25% or >31% as defining biochemical thiamin deficiency were likewise not useful for predicting infantile beriberi (OR 1.77, 95%CI 0.68–4.63 and OR 2.1, 95%CI 0.75–5.96, respectively).

**Figure 3:**
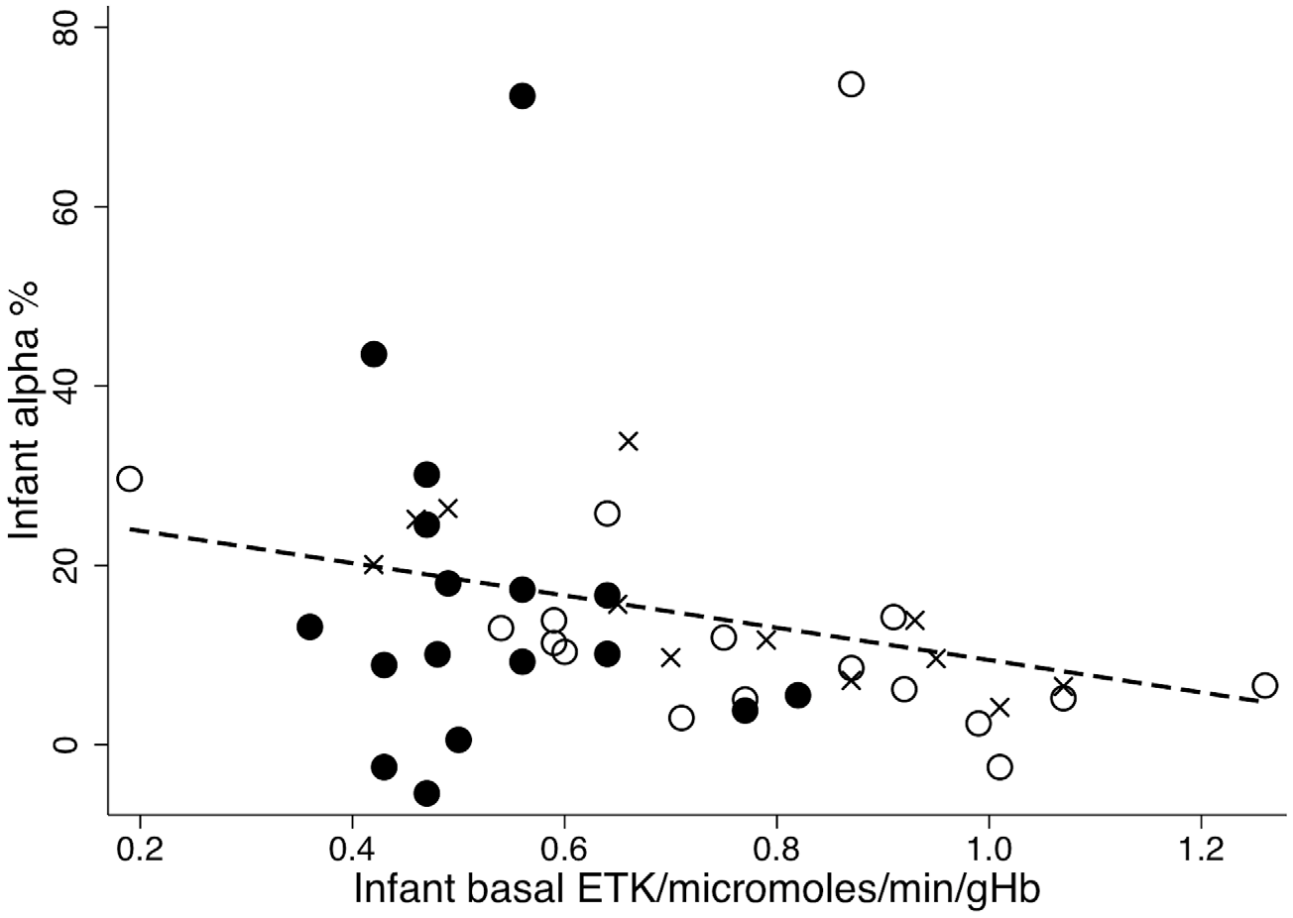
Relationship between infant basal ETK and infant α. Black circles = infants with beriberi (n = 17), crosses = infant afebrile controls (n = 12), open circles = infant febrile controls (n = 17). Spearman rho = −0.4219, P = 0.004, Infant basal ETK = 27.46−18.02* infant α.

**Figure 4:**
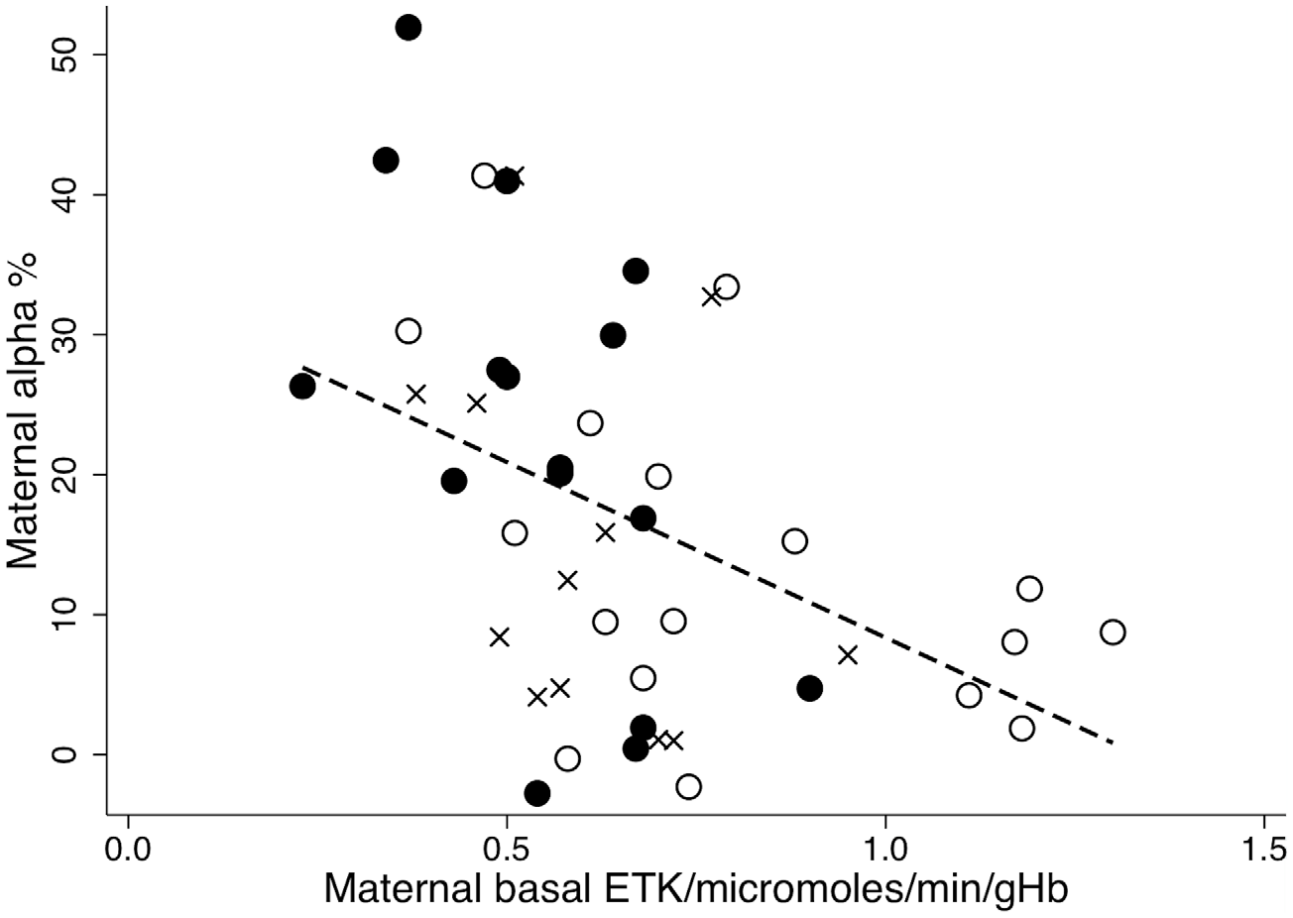
Relationship between maternal basal ETK and maternal α. Black circles = infants with beriberi (n = 17), crosses = infant afebrile controls (n = 12), open circles = infant febrile controls (n = 17). Pearson's r = −0.4359, P = <0.003, Maternal basal ETK = 33.43−25.06*Maternal α.

Maternal troponin T could not be compared as only one mother had detectable troponin T. The relationship between infant basal ETK and **α** is shown in Fig 3–infants with beriberi clustering at low basal ETK but with a wide variation in **α**. In contrast, maternal **α** tended to be higher at lower maternal basal ETK (Pearson's r = −0.4359, p = 0.003) (Fig. 4).

### Association of clinical features with infantile beriberi

All infants with clinical beriberi were breastfed and therefore this variable could not be evaluated as a potential confounder or predictor. Two variables differed between afebrile and febrile control infant-mother pairs–the mothers of febrile infants had less schooling (P = 0.0001) and less commonly observed food avoidance (P = 0.004) (Tables 1 & 2) than the mothers of afebrile infants.

Six clinical variables were significantly associated (p≤0.02) with beriberi in the univariate analyses (Table 1 & 2): mother was a rice farmer, had fewer years of schooling (in comparison to afebrile infants only), observed food avoidance (in comparison to febrile infants only), infant was not bottle fed, had a sibling who died when <1 year old, and whose father was a rice farmer. However, only two independent predictors of beriberi were identified by the multivariate model: that father was a rice farmer (OR 4.7, 95%CI 1.92 to 11.4) and that the mother observed food avoidance (OR 6.25, 95%CI 1.86 to 21.0). These two independent predictors were then assessed in three separate models with detectable plasma troponin T, infant basal ETK<59 micromoles/min/gHb, and maternal basal ETK as additional independent variables. As the relationship between clinical beriberi and basal ETK was stronger than with activated ETK, basal ETK only was used. Detectable plasma troponin T and infant basal ETK<59 micromoles/min/gHb remained significant independent predictors for infantile beriberi (OR 4.3, 95%CI 1.25 to 15.0, adjusted for mother observed food taboos, and OR 15.9, 95%CI 2.03 to 124.2, for detectable troponin T and ETK<59 micromoles/min/gHb, respectively).

## Discussion

Lao people have multiple risk factors for thiamin deficiency. The consumption of mechanically polished rice, alcohol and thiaminase-containing foods such as ‘paa dek’ (fermented fish paste), thiamin antagonists such as betel nut, and the hard physical labour of rural rice farming are likely to be important in adults [4], [47], [48]. Traditional prolonged post-partum Lao maternal food avoidances may lead to fatal wet beriberi in infants and neurological symptoms in nursing mothers. In Vientiane, 93% of women stated that they avoided foods, such as pork, diverse vegetables and fruit, in the first 2–3 months after delivery and 97% of post-partum women were estimated to have an inadequate thiamin intake [48]. Malaria and other febrile episodes in pregnant and post-partum women may predispose to beriberi in their infants by further depleting short lived body stores [35], [39]. A case control study [15] at Mahosot Hospital, suggested that, compared with control mothers, mothers of infants with beriberi had significantly less diet diversity, soaked glutinous rice significantly longer or were more likely to pour off excess water from non-glutinous rice, had fewer years of schooling, were more likely to report that income was inadequate for basic needs, to perform hard physical labour and to be married to farmers.

Infantile beriberi is a lethal condition if unrecognized and untreated. This study strongly suggests that basal ETK, and to a lesser extent activated ETK, and not the α coefficient is the best biochemical marker of infantile beriberi. The results also suggest that markers of cardiac dysfunction, such as troponin T, may be useful surrogate markers for infantile beriberi.

The study has important limitations and a prospective assessment with a larger sample size is needed. Some clinical and laboratory data collected for cases were not collected for controls. Detailed records of infant-mother pairs not recruited to the study were not kept so we are unable to compare cases and controls with those admitted but not recruited. Two laboratories had to be used for ETK determinations and haemoglobin concentrations were not assayed at one and therefore basal and activated ETK in micromoles/min/gHb could not be expressed for these samples. We did not compare the enzymatic assays with high performance liquid chromatography methods for thiamin diphosphate determination [3]. Due to the expense of the assays, ntBNP determinations were performed only on a subset. Controls were representative of infants who would have been selected as cases if they had clinical beriberi. However, although none of the controls had overt beriberi, they may have had subclinical thiamin deficiency [19] and therefore blurred the distinction between cases and controls. As fever may precipitate beriberi [4], [35], [39] excluding fever from the case definition of beriberi, as we have done, may have limited our description as infants with concurrent sepsis and beriberi are likely to occur.

Basal ETK is influenced by factors other than thiamin status. Diabetes and liver dysfunction may reduce basal ETK [49] whilst B12 deficiency may increase basal ETK [50]. Younger red blood cells have higher basal ETK and differences between patients could reflect variation in haematopoesis and red cell survival [51]. Hypomagnesaemia may reduce α [52].

In agreement with Soukaloun *et al.* [15], we also found, on univariate analysis, that infantile beriberi was associated with mothers who were farmers and had less schooling (but only for afebrile controls). The gradient of the relationship between maternal and infant basal ETK (Fig 2), with the intercept on the y-axis, suggests that thiamin is preferentially concentrated in the infant rather than the mother, consistent with the findings of Migasena *et al.* [31].

These data suggest that markers of cardiac dysfunction, such as plasma troponin T, could be useful surrogate markers in the diagnosis of infantile beriberi. Troponin T is a 39-kDa subunit of the thin filaments of the contractile apparatus of the myocardium. Tran [53] described two alcoholic adults with cardiac dysfunction and suspected beriberi with raised Troponin I, another subunit of the troponin complex, who improved with thiamin therapy. The pathophysiology of cardiac dysfunction in infantile beriberi is not understood and, as far as we are aware, there have been no echocardiographic or cathertisation studies. In Lao infants troponin T, BNP or ntBNP may also be raised in cardiac ischaemia, contusion, unstable congenital heart disease, myocarditis, rheumatic fever, Kawasaki disease and due to cardiac damage secondary to, for example, asphyxia and septicaemia [33], [44]. Further examination of a diversity of biochemical markers of cardiac dysfunction and echocardiography may provide insights into the pathophysiology of infantile beriberi and allow firmer conclusions on the most appropriate biochemical surrogate markers. The development of quantitative bedside rapid tests for troponin T and ntBNP [33], [43] raises the possibility that they may be appropriate rapid, accessible screening tests for infantile beriberi.

The data presented here and those of Soukaloun *et al.* [15] and Khounnorath *et al.* [19] suggest that infantile beriberi is an important cause of morbidity and mortality in Vientiane. There are anecdotal reports of beriberi from other areas of Laos (C. Perks, G. Slesak, L. Srour, H. Barennes, T. Saito pers. comm.) but, given the wide ethnic and nutritional diversity in Laos, whether all communities are afflicted remains very unclear [12]. Comprehensive urgent investigation of the variability of thiamin content of glutinous rice, in relation to milling, soaking and cooking practices, post-partum food avoidance behaviour in relation to ethnic and geographical variability, and estimation of the burden of thiamin deficiency on infant mortality is needed. The proportion of infants dying at 2–4 months of age may be useful rapid indicator of the public health importance of beriberi in a community, as was noted many decades ago [5], [19]. Given the difficulties in venesection of young infants, this study also suggests that post-partum maternal basal ETK may provide a rapid biochemical screen of the thiamin status of a community's infants.

## Supporting Information

Checklist S1 STARD Checklist
(0.05 MB DOC)
